# Carious Teeth and Tuberculosis

**Published:** 1897-07

**Authors:** 


### Carious Teeth and Tuberculosis.

Strack (*American Journal of the Medical Sciences*) has investi gated the possible relation of carious teeth to tuberculosis, with valuable and suggestive results. Among 113 children with enlarged cervical glands none of the ordinarily accepted causes could be traced in 41 percent, and attention was called to the co-existence of carious teeth. The enlarged glands nearly always correspond in position to the affected teeth. In many cases toothache had preceded the enlargement, or the caries was evidently primary. In two cases positive evidence of the relationship of the disease was adduced. These were a boy of eighteen years and a girl of fourteen years, both healthy previously, and with no family history of tuberculosis. Enlargement of the cervical glands followed toothache. In the first case tubercle bacilli were found in the carious molar teeth; in the other a suspicious-looking granulation was found between the roots of a molar, which, on section, showed tubercles with giant cells.

